# Omni-Refinement Attention Network for Lane Detection

**DOI:** 10.3390/s25196150

**Published:** 2025-10-04

**Authors:** Boyuan Zhang, Lanchun Zhang, Tianbo Wang, Yingjun Wei, Ziyan Chen, Bin Cao

**Affiliations:** 1 School of Automible and Traffic Engineering, Jiangsu University of Technology, Changzhou 213001, China; zhang572236@163.com (B.Z.); wtbnjust@126.com (T.W.); czy173617@163.com (Z.C.); cb20011106@163.com (B.C.); 2 School of Vehicle Engineering, Chongqin University of Technology, Chongqin 400054, China; weiyingjun71@sina.com

**Keywords:** lane detection, autonomous driving, attention mechanism

## Abstract

Lane detection is a fundamental component of perception systems in autonomous driving. Despite significant progress in this area, existing methods still face challenges in complex scenarios such as abnormal weather, occlusions, and curved roads. These situations typically demand the integration of both the global semantic context and local visual features to predict the lane position and shape. This paper presents ORANet, an enhanced lane detection framework built upon the baseline CLRNet. ORANet incorporates two novel modules: Enhanced Coordinate Attention (EnCA) and Channel–Spatial Shuffle Attention (CSSA). EnCA models long-range lane structures while effectively capturing global semantic information, whereas CSSA strengthens the precise extraction of local features and provides optimized inputs for EnCA. These components operate in hierarchical synergy, collectively establishing a complete enhancement pathway from refined local feature extraction to efficient global feature fusion. The experimental results demonstrate that ORANet achieves greater performance stability than CLRNet in complex roadway scenarios. Notably, under shadow conditions, ORANet achieves an F1 score improvement of nearly 3% over CLRNet. These results highlight the potential of ORANet for reliable lane detection in real-world autonomous driving environments.

## 1. Introduction

Lane detection is a core component of autonomous driving perception and is thus fundamental to the operation of Advanced Driver Assistance Systems (ADASs) [1], such as Lane Keeping Systems (LKSs) [2] and Adaptive Cruise Control (ACC) [3]. Owing to its potential to improve road traffic safety and advance the development of autonomous driving and intelligent transportation systems, research on lane detection technology possesses significant academic value and broad applicability.

In order to adapt to varying road and lighting conditions, most traditional lane detection methods rely on manually tuned parameters, such as HSL color mapping [4] and the Hough Transform [5], rendering them highly susceptible to external interference. However, these methods exhibit limited generalization and scalability in complex roadway environments due to their reliance on extensive rule-based design and parameter configuration.

Deep learning-based lane detection methods have achieved substantial progress following the rapid advancement of artificial intelligence in recent years. These approaches can automatically extract lane features from images [6], exhibiting stronger modeling capabilities and feature learning performance than traditional algorithms.

**Figure 1:**
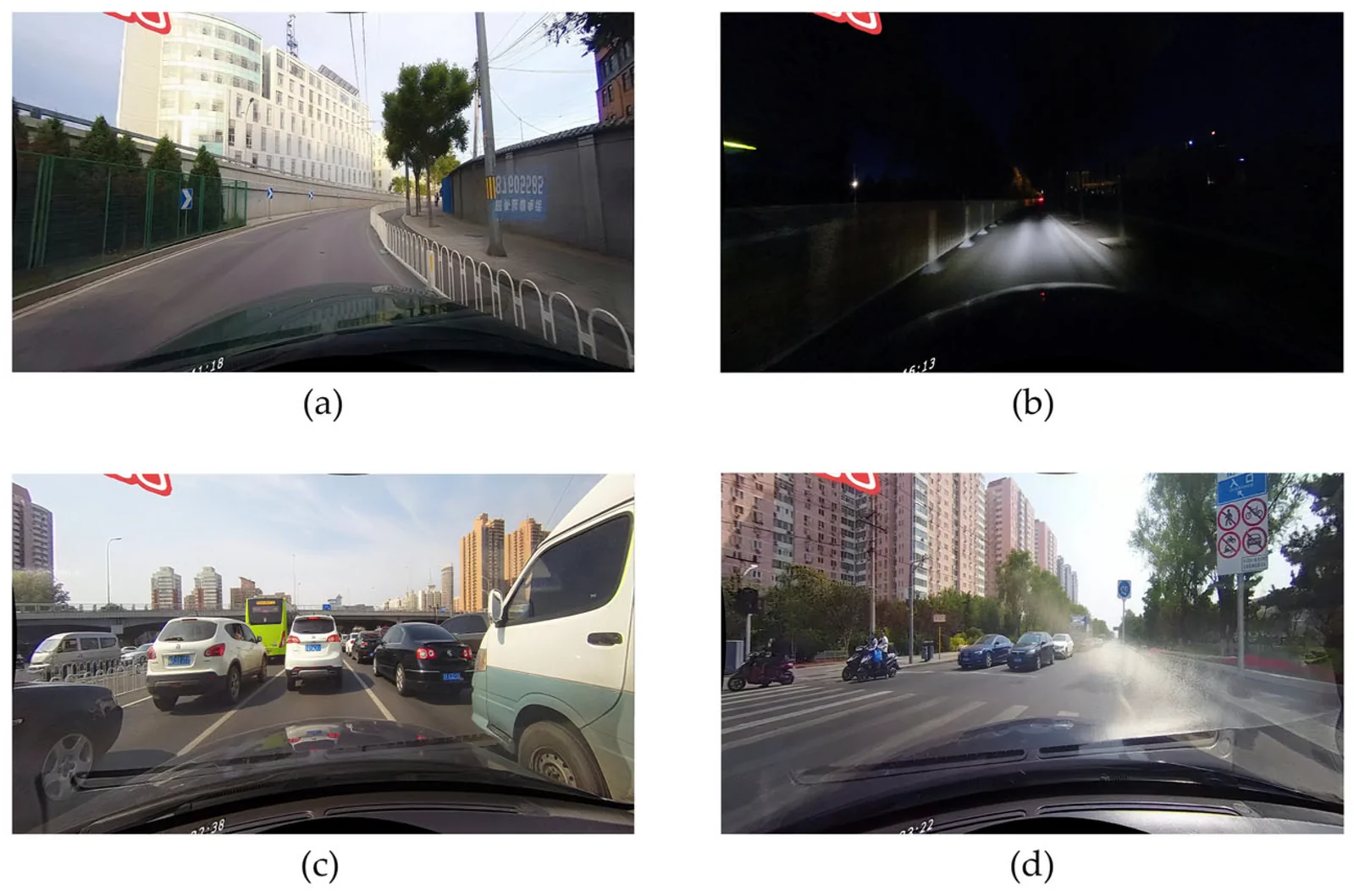
Some challenging scenarios for lane detection. (**a**) Curve; (**b**) night; (**c**) lanes occluded by other vehicles; (**d**) lanes obstructed by glare.

Most deep learning-based lane detection methods are based on segmentation- or anchor-based object detection techniques and have achieved considerable success [7]. Nevertheless, these methods still face significant challenges under extreme conditions, as illustrated in Figure 1.

As an example, UFLD [8] employs global features with large receptive fields to handle complex road scenes; however, it suffers from suboptimal detection accuracy resulting from missing key information when capturing fine-grained lane structures, limited ability to capture long-range contextual information, and insufficient sensitivity to local details. CLRNet [9] enhances lane detection performance by incorporating richer contextual information, thus achieving relatively higher accuracy, and yet still faces limitations in global feature fusion and lacks effective local feature extraction capabilities. Such deficiencies pose substantial challenges to the practical application of lane detection techniques.

Notably, recent progress in related fields has inspired approaches that address analogous challenges. For example, Tao [10] addressed micro-object detection in industrial scenarios by introducing a Receptive Field Attention mechanism coupled with multi-scale feature fusion. Such methods offer useful guidance for improving lane detection performance under occlusion and complex scene conditions.

Therefore, achieving accurate lane detection in complex scenarios depends on developing a more effective feature fusion strategy, as supported by precise and refined feature extraction techniques. In response, we propose ORANet, a novel lane detection framework built upon the original CLRNet architecture. ORANet incorporates two meticulously designed components: Enhanced Coordinate Attention (EnCA) and Channel–Spatial Shuffle Attention (CSSA). EnCA employs directional adaptive pooling and cross-layer feature fusion mechanisms to accurately model the directional long-range structure of lanes while efficiently integrating global semantic information and thereby significantly improving the quality of feature fusion. CSSA utilizes a channel–spatial collaborative attention mechanism to strengthen the semantic discriminability and spatial continuity perception in low-level features, thus providing high-quality input support for EnCA’s global feature fusion. Through hierarchical collaboration of the two, ORANet significantly improves the accuracy and robustness of lane detection under complex scenarios. Our main contributions can be summarized as follows:

1. We propose a novel feature fusion enhancement module (EnCA), which improves fusion quality through directional adaptive pooling and cross-layer integration.
2. We design an innovative feature extraction enhancement module (CSSA) that strengthens local representations via coordinated channel–spatial attention.
3. We conduct comprehensive experiments to validate the effectiveness of ORANet, achieving state-of-the-art results across multiple benchmark datasets.

## 2. Related Work

Deep learning-based lane detection methods can be broadly classified into four categories according to their lane representations:

### 2.1. Segmentation-Based Methods

This method treats lane detection as a pixel-level segmentation problem, where every pixel is categorized as either part of a lane or the background. Early versions of SCNN [11] framed lane detection as a multi-category instance segmentation problem, introducing a spatial CNN to learn shape priors. To meet real-time application requirements, ENet-SAD [12] proposed an innovative knowledge distillation strategy—Self-Attention Distillation (SAD)—which was integrated into ENet’s [13] encoder to enhance shallow feature representations by leveraging rich information from deeper layers. Subsequent research has further explored optimization strategies; for example, LaneSegNet [14] combined hybrid attention mechanisms with parallel multi-scale feature extraction to enlarge the receptive field and enhance task-specific focus, and AF-ICNet [15] extended this line of work by introducing small-target category attention and feature fusion strategies into semantic segmentation for unstructured scenes. Despite these advances, segmentation-based lane detection methods still suffer from high computational costs and limited robustness under occlusion or other extreme conditions.

### 2.2. Row-Wise-Based Methods

Lane detection approaches based on row-wise processing focus on optimizing computational efficiency while ensuring precise lane shape prediction. UFLD [8] formulates lane detection as a row-wise classification perspective utilizing global features, which significantly accelerates inference speed. In its subsequent version, UFLD v2 [16], a hybrid anchor system, was introduced to reduce localization errors; however, UFLD [8] consistently overlooks the influence of tilt angles in the loss functions. CondLaneNet [17] employs dynamic convolution on feature maps to perform simultaneous row-wise classification and x-coordinate regression for lane localization. Although these methods fundamentally rely on identifying lane starting points, they often struggle in complex scenarios where such points are ambiguous or occluded, resulting in degraded performance.

### 2.3. Keypoint-Based Methods

Motivated by advances in human pose estimation [18], recent studies have reformulated lane detection to focus on keypoint detection and their association as follows: PINet [19] employs stacked hourglass networks to predict keypoint locations and feature embeddings, clustering lane instances by measuring embedding similarities; FOLOLane [20] generates full-resolution pixel-wise heatmaps to detect keypoints and introduces local geometric constraints to associate points within the same lane; GANet [21] leverages global contextual information for keypoint prediction by regressing each point to its parent lane, as well as utilizing a Lane-Aware Feature Aggregator to capture local correlations between adjacent keypoints; and CANet [22] utilizes U-shaped guiding curves to acquire instance keypoints and infers lane point sets through an adaptive decoder. Although keypoint-based methods provide fine-grained lane representations, they typically require costly post-processing steps to reconstruct full lane instances, limiting their computational efficiency in real-world applications.

### 2.4. Anchor-Based Methods

Anchor-based lane detection methods, analogous to YOLO [23,24], employ predefined line anchors as references to accurately regress lane positions. These methods typically apply Non-Maximum Suppression (NMS) [25] to eliminate redundant or erroneous anchor predictions and generate final outputs. Line-CNN [26] pioneered the application of line anchors in lane detection, while LaneATT [27] recognized the significance of global semantics and proposed an anchor-based attention mechanism to enhance detection accuracy. CLRNet [9] further refined this approach by discretizing anchors into 72 points to strike a balance between detection performance and inference speed while incorporating richer contextual features. However, reliance on predefined anchors constrains the adaptability of these methods, presenting challenges across diverse extreme scenarios.

In addition to algorithmic advances, new datasets have been developed to better reflect challenging real-world conditions. For example, Zunair introduced RSUD20K [28], a large-scale benchmark comprising over 20K images and 130K annotations from Bangladeshi roads, which complements widely used datasets such as Cityscapes, KITTI, and BDD100K. To provide a clearer overview of existing lane detection approaches, we summarize representative methods in Table 1. The comparison covers datasets, model architectures, feature extraction strategies, evaluation metrics, and validation outcomes. This structured review highlights the strengths and weaknesses of different paradigms and provides a foundation for understanding the motivation behind our proposed ORANet.

As shown in Table 1, existing methods differ significantly in datasets, backbone networks, and feature extraction designs. Segmentation-based approaches achieve fine-grained predictions but suffer from high computational costs; row-wise and keypoint-based methods emphasize efficiency or precision but face robustness issues in complex environments; anchor-based methods balance speed and accuracy but struggle with long-range structural modeling. Furthermore, evaluation protocols remain dataset-specific, limiting generalization. These observations collectively underscore the need for a unified framework that can simultaneously refine local visual details and integrate global semantic contexts—a gap that ORANet aims to fill.

## 3. Approach

**Figure 2:**
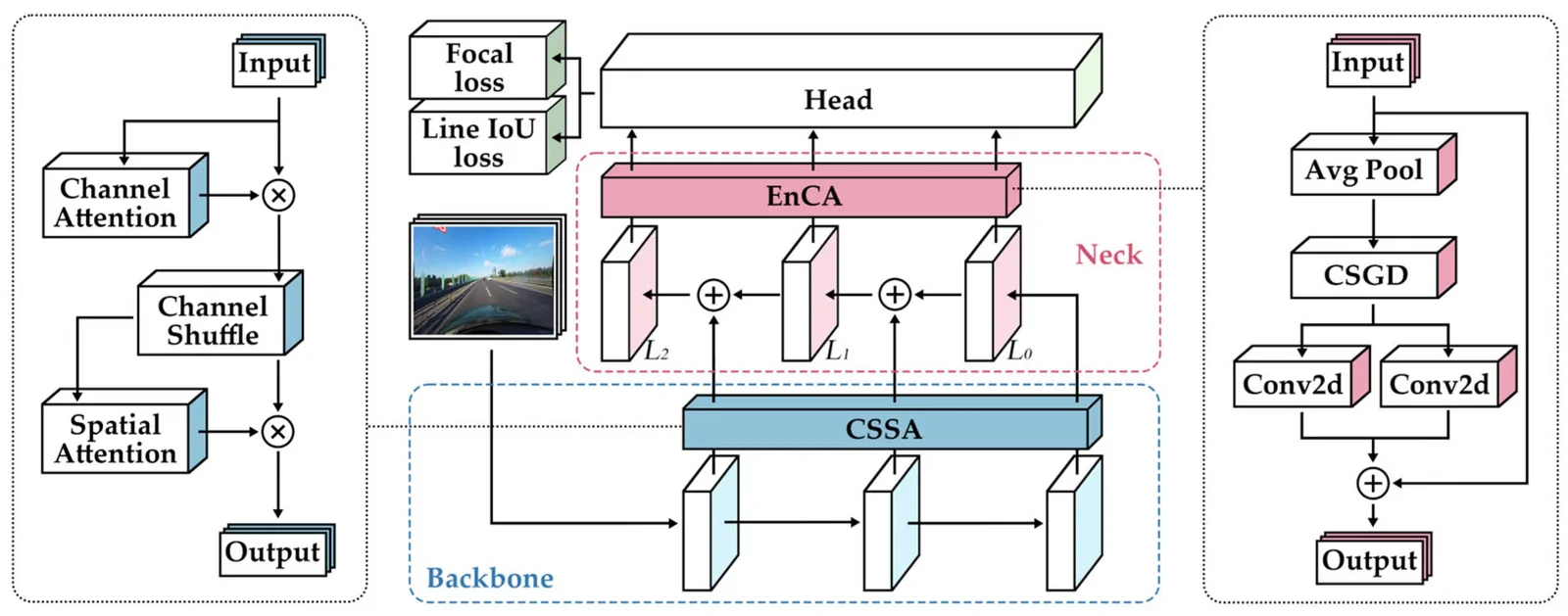
Overall structure of the ORANet.

Building upon the limitations identified in existing lane detection paradigms, we propose ORANet, a novel anchor-based framework designed to enhance both accuracy and robustness in complex scenarios. The overall architecture of ORANet is illustrated in Figure 2. Specifically, the backbone is built upon ResNet-18 [29], a lightweight yet effective CNN architecture widely used in lane detection tasks. To enhance its feature representation capacity, we integrate the CSSA module into the backbone, which further improves semantic expressiveness and spatial sensitivity and thus refines feature extraction. The extracted multi-level features are then fed into a Feature Pyramid Network (FPN) [30] to generate multi-scale representations, which are subsequently enhanced by the proposed EnCA module to strengthen cross-scale fusion by capturing long-range lane structures and global contextual dependencies. Finally, the fused multi-scale features are passed to the detection head, which is jointly supervised by Focal Loss and Line IoU Loss [9], enabling the network to produce accurate and robust lane detection results, even under challenging conditions.

**Figure 3:**
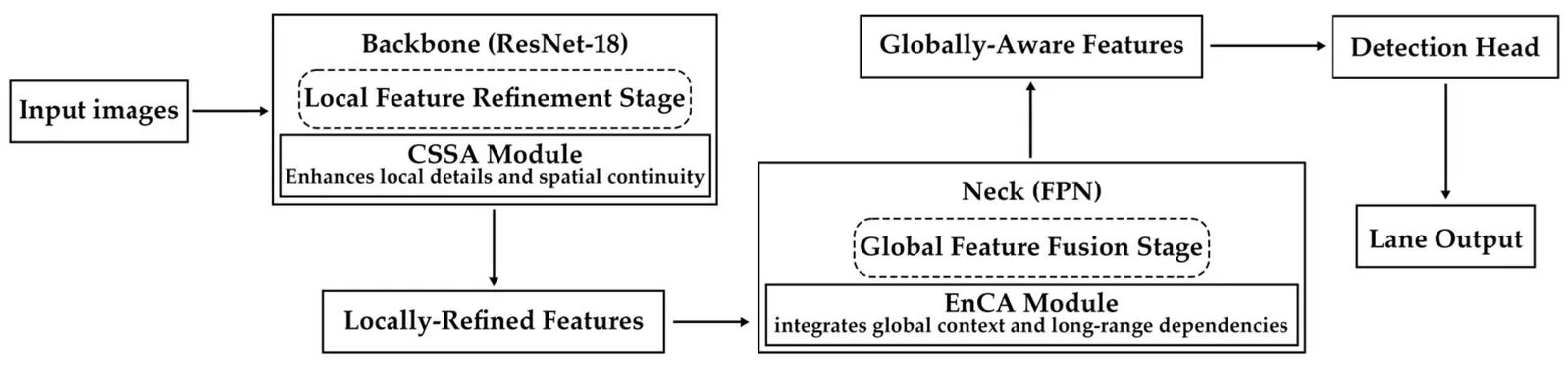
Flowchart of the ORANet.

To clarify the hierarchical synergy between local visual detail and global semantic context within ORANet, Figure 3 presents a conceptual flowchart of the feature processing pathway. The framework is designed to first refine local features before integrating them into a global context, establishing a comprehensive enhancement process.

### 3.1. Enhanced Coordinate Attention (EnCA)

In certain CNN-based lane detection methods [12,26,29,31], the FPN exhibits notable limitations in effectively modeling the slender spatial structure of lane lines and fusing cross-layer global semantics. However, accurate lane localization in complex scenarios requires the FPN to capture intrinsic directional long-range dependencies while enriching low-level details with high-level semantic information; such shortcomings notably degrade the model’s robustness and performance when faced with complex environmental conditions.

To achieve a more efficient architecture with stronger directional awareness and cross-level information guidance, we introduce a novel module termed EnCA, whose design is motivated by the geometric prior of lane lines which, in image space, predominantly extend along horizontal or vertical directions, demonstrating pronounced directional continuity and long-range structural properties. Unlike generic approaches such as deformable convolutions, directional pooling aggregates features in a structured manner along orthogonal axes, thereby explicitly embedding directional inductive bias and enabling the model to capture global spatial dependencies of lane lines more effectively while simultaneously preserving computational efficiency.

Specifically, EnCA leverages directional adaptive pooling—separately processing horizontal and vertical orientations—and a cross-layer feature fusion mechanism to significantly improve the FPN’s ability to capture spatial structures and global context, resulting in richer and more discriminative feature representations for the detection head. EnCA incorporates depth-wise separable convolutions to reduce the number of parameters and computational cost while retaining its effectiveness in enhancing spatial–semantic feature representations essential for fine-grained lane detection, thus maintaining a favorable trade-off between accuracy and efficiency.

**Figure 4:**
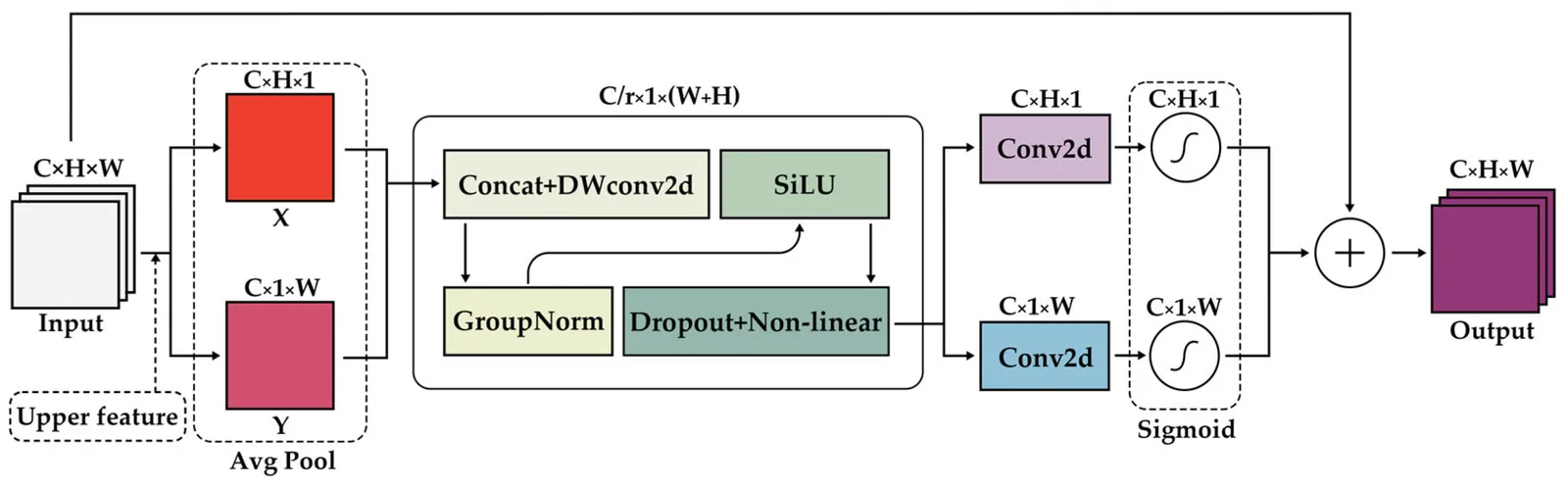
Detailed structure of the EnCA.

As illustrated in Figure 4, given the input feature map $F_{in}$ and the high-level semantic feature map $F_{upper}$, EnCA first applies adaptive average pooling to Fupper to spatially align it with $F_{in}$; the two are then merged via concatenation along the channel dimension to incorporate richer contextual cues, formulated as

(1) $$F_{cat}=Concat\left(F_{in},AdaptiveAvgPool\left(F_{upper}\right)\right)$$

Following the incorporation of enriched feature information, to effectively capture lane continuity across orientations, the concatenated feature map $F_{cat}$ undergoes coordinate-wise global pooling along two orthogonal axes. This operation generates horizontal- ($F_{h}$) and vertical-direction ($F_{w}$) features, where $F_{w}$ is transposed before concatenation to ensure spatial alignment. The two directional features are then fused along the spatial dimension to produce the direction-aware representation $F_{dir}$, as defined by

(2) $$F_{dir}=Concat\left(F_{h},F_{w}^{⊺}\right)$$

The feature map $F_{dir}$ is passed through a lightweight convolutional pathway composed of a series of feature compression and transformation operations to compress redundant dimensions and refine directional representations. In particular, the operation begins with a 1 × 1 point-wise convolution, which is subsequently followed by a depth-wise separable convolution (DWConv). The output is then sequentially processed with Group Normalization (GN), the SiLU activation function **σ**, and Dropout, with the aim of improving training stability and enhancing the model’s generalization capability:

(3) $$Z=Dropout(\sigma \left(GN\left(DWconv\left({Conv}_{1\times 1}\left(F_{dir}\right)\right)\right)\right))$$

Following the splitting of Z into horizontal and vertical branches, each branch undergoes convolutional processing to generate corresponding attention weights $A_{h}$ and $A_{w}$, which are then applied to the original input feature map $F_{in}$, achieving direction-aware feature enhancement through a residual connection:

(4) $$F_{out}=F_{in}+F_{in}⊙A_{h}⊙A_{w}$$

### 3.2. Channel Spatial Shuffle Attention (CSSA)

Although the EnCA module effectively improves spatial structure modeling and global semantic integration within the FPN hierarchy, the overall performance of FPN remains heavily dependent on the quality of features extracted by the backbone network. However, the original CLRNet backbone exhibits notable limitations in this regard: (1) conventional convolutions fail to adequately capture inter-channel dependencies, leading to uncalibrated semantic feature responses, and (2) their inherently local receptive fields constrain the acquisition of long-range spatial context, which is essential for accurately modeling continuous lane structures. These deficiencies in low-level representations cannot be fully remedied, even with an EnCA-enhanced FPN.

Existing attention mechanisms like SE and CBAM have been employed to address such limitations in the backbone [32,33]. The SE module focuses solely on channel re-weighting while entirely overlooking spatial context, while CBAM improves the process by sequentially combining channel and spatial attention, yet its design still lacks explicit cross-channel interaction and sufficient capacity for capturing long-range spatial dependencies. These inherent constraints prevent them from fully resolving the feature representation issues in lane detection backbones.

To overcome this, we integrate the CSSA module into key stages of the backbone. CSSA is designed to complement EnCA in a hierarchically coordinated manner: during early-stage feature extraction, it enhances critical semantic channels via explicit channel attention and facilitates inter-channel interactions through channel shuffle operations. Furthermore, a large-receptive-field spatial attention mechanism captures long-range spatial dependencies; this joint channel–spatial enhancement strategy equips the backbone with more discriminative and structured features, which in turn strengthen the global semantic fusion capabilities of EnCA in later FPN stages. Collectively, EnCA and CSSA construct a comprehensive enhancement pathway spanning from local feature extraction to global feature fusion.

**Figure 5:**
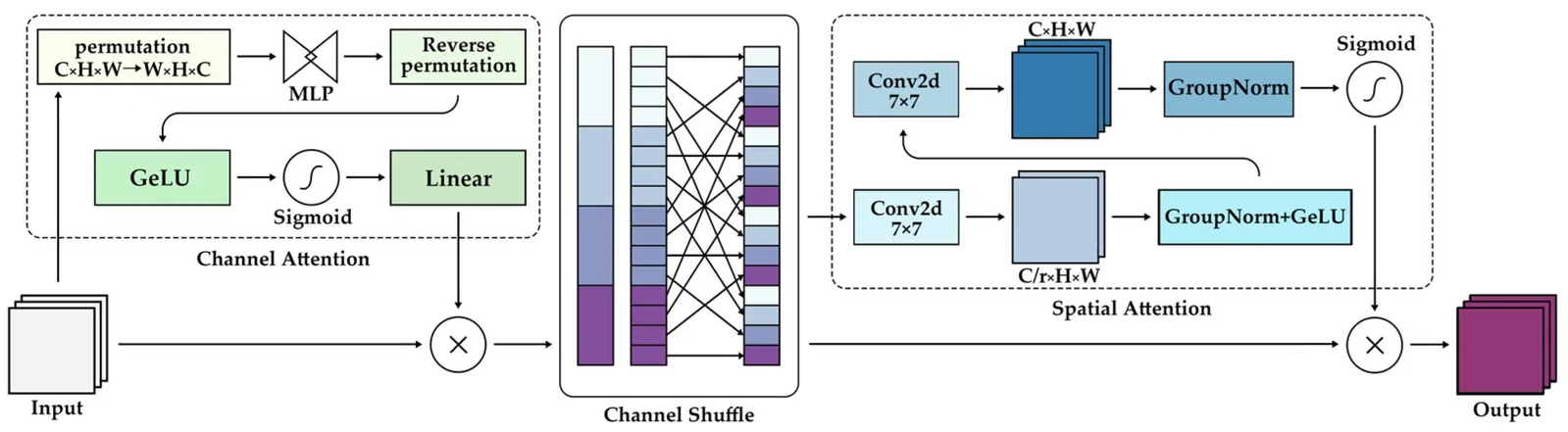
Detailed structure of the CSSA.

In conventional convolutional neural networks, inter-channel dependencies are frequently neglected despite their crucial role in capturing global contextual information, which may lead to suboptimal utilization of feature representations and hinder comprehensive global feature extraction. To overcome this limitation, the channel attention submodule within CSSA explicitly models inter-channel relationships, as illustrated in Figure 5, specifically via the input feature map $F_{in}$ through dimension transposition from **C × H × W** to **W × H × C**. This transposed tensor is then passed through a two-layer multilayer perceptron (MLP): the first layer reduces the channel dimension to **C/4**, followed by a GeLU activation for non-linear transformation, while the second layer restores the original dimensionality. After processing, the output is inversely permuted back to **C × H × W**, and a sigmoid activation σ is applied to generate the channel attention map. The final attention map is multiplied element-wise with the original input to yield the enhanced feature representation $F_{channel}$:

(5) $$F_{channel}=\sigma (MLP\left(Permute\left(F_{in}\right)\right))⊙F_{in}$$

Despite the incorporation of channel attention, insufficient inter-channel information interactions may still limit the discriminative power of the feature representations, thereby weakening the overall effectiveness of the attention mechanism. A channel shuffle operation is introduced to mitigate this issue, facilitating cross-channel communication and enhancing feature diversity. Specifically, the attention-refined feature map $F_{channel}$ is first partitioned into four groups along the channel dimension, each comprising **C/4** channels; within each group, a transposition-based permutation is applied to rearrange the channels, effectively promoting interaction across previously isolated channel groups. The permuted features are then reshaped back to the original spatial dimensions to produce the shuffled feature map $F_{shuffle}$:

(6) $$F_{shuffle}=ChannelShuffle(F_{channel})$$

Relying solely on channel attention and shuffle operations, however, risks underutilizing spatial information, which is equally vital for capturing both local and global features. Neglecting spatial dimensions may result in the model missing critical details embedded in feature maps. To address this, the spatial attention submodule processes the input feature $F_{shuffle}$ through a 7 × 7 convolutional layer that reduces the channel dimension to **C/4**, followed by Batch Normalization (BN) and a GeLU activation for non-linear transformation; a second 7 × 7 convolutional layer subsequently restores the channel dimension to **C**, followed by another BN layer. The spatial attention map is generated by applying a sigmoid activation σ. Finally, element-wise multiplication between this spatial attention map and $F_{shuffle}$ produces the refined output feature map $F_{out}$:

(7) $$F_{out}=\sigma (Conv\left(GN\left(GeLU\left(Conv\left(F_{shuffle}\right)\right)\right)\right))⊙F_{shuffle}$$

## 4. Experiments

### 4.1. Datasets

This study conducts evaluations on two widely used benchmark datasets for lane detection, namely CULane [11] and TuSimple [34].

**Figure 6:**
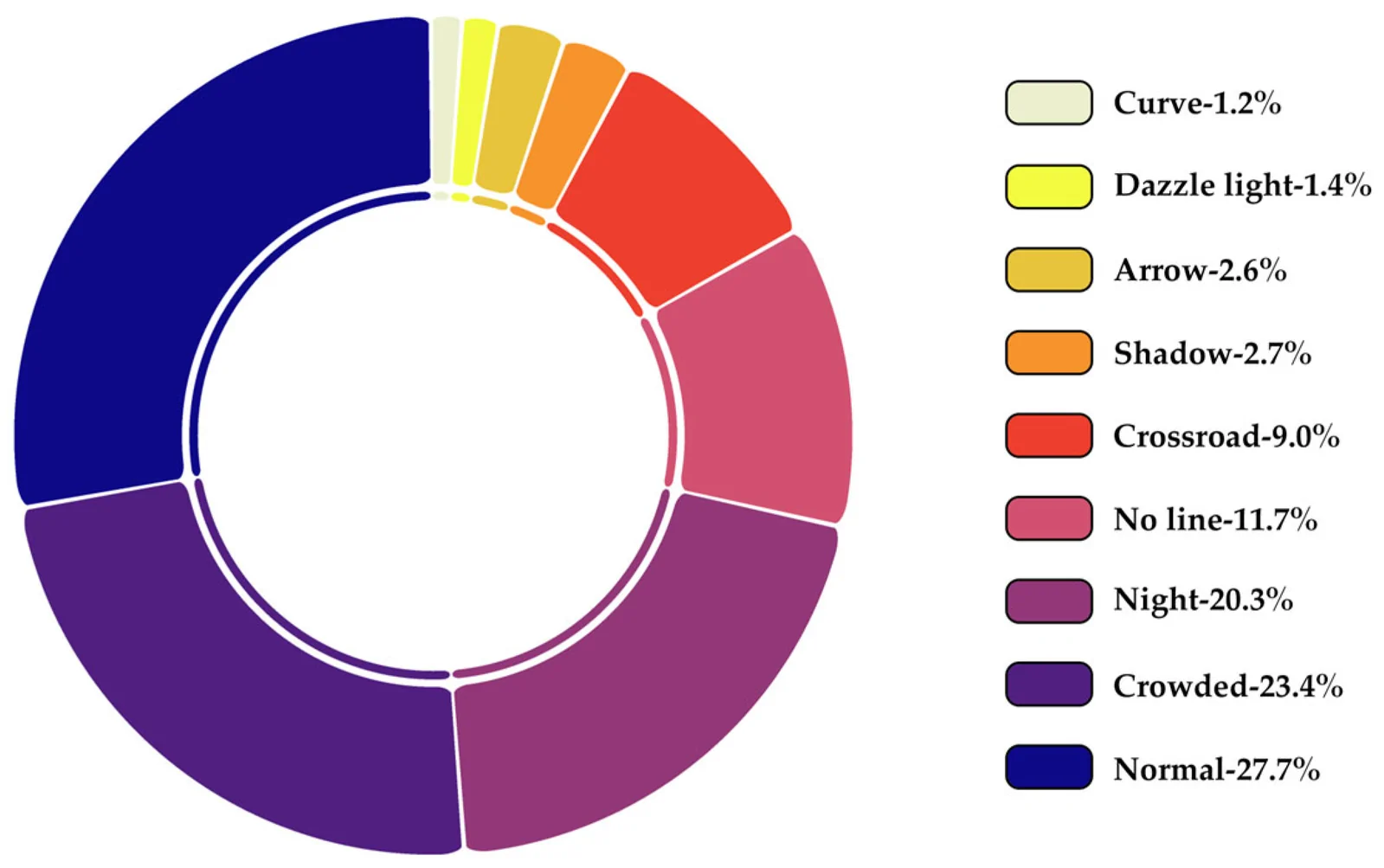
The Scene Composition of the CULane Datasets.

CULane is a large-scale and highly challenging dataset extensively utilized in autonomous driving and intelligent transportation research. It comprises 133,235 images acquired through camera systems deployed on six individual vehicles driving through various road environments in Beijing; the dataset covers a wide range of urban and suburban traffic scenarios, with all frames standardized to a resolution of 1640 × 590 pixels. It is then divided into training, validation, and testing subsets, subsequently being further categorized into nine challenging scenarios—such as crowded scenes, curves, and shadows—to assess model robustness under diverse conditions. The detailed scene distribution is illustrated in Figure 6.

In contrast, TuSimple focuses solely on highway scenarios, primarily captured under clear weather conditions. It contains 3268, 358, and 2782 training, validation, and test images, respectively, all with a resolution of 1280 × 720 pixels. Despite its relatively constrained domain, TuSimple remains a widely accepted benchmark for evaluating baseline lane detection performance.

### 4.2. Implementation Details

Experimental details align with the baseline CLRNet [9] to facilitate parameter comparison and tuning; specifically, we adopt a ResNet backbone [29] pretrained on ImageNet and implemented using PyTorch (v2.4.1). All input images are resized to 800 × 320 pixels for training and evaluation. For model optimization, we selected the AdamW optimizer [35], chosen for its superior handling of weight decay, which typically improves model generalization. We configured it with standard hyperparameter values: a weight decay of 0.01, β1 = 0.9, and β2 = 0.999. This optimizer was paired with a cosine annealing learning rate schedule [36] for progressive decay during training. While the original CLRNet [9] adopts a learning rate of 0.0006, we performed extensive hyperparameter tuning within the range of 0.0001–0.001. In Table 2, experimental results suggest that a learning rate of 0.0003 offers the best balance between training stability and final accuracy in our implementation.

During initial training, we followed baseline settings with 15 and 70 epochs for CULane [11] and TuSimple [34], respectively. The number of training epochs was adaptively selected based on the convergence of loss and the F1-score, ensuring sufficient optimization without overfitting. We incorporated data augmentation techniques, including random pixel dropout and cutout regularization, to improve robustness under diverse road conditions and reduce overfitting risks. These choices were informed by established practices in prior studies and were further validated through empirical testing.

All experiments were conducted on Windows Subsystem for Linux 2.0 (WSL 2.0), utilizing an NVIDIA GeForce RTX 5070 Ti GPU and an Intel^®^ Core™ i7-14700KF CPU. All models were developed and executed in a Python 3.8 runtime environment.

### 4.3. Evaluation Metrics

For the CULane dataset [11], we adopt the F1-score as the primary evaluation metric. A predicted lane is regarded as a true positive (TP) if its Intersection over Union (IoU) with the ground truth exceeds a predefined threshold; otherwise, it is categorized as a false positive (FP) or false negative (FN). The F1-score is computed as

(8) $$F_{1}=\frac{2\times Precision\times Recall}{Precision+Recall}$$

where precision and recall are defined by

(9) $$precision=\frac{TP}{TP+FP},\;Recall=\frac{TP}{TP+FN}$$

In addition, we follow CLRNet [9] by adopting two specific IoU thresholds—0.5 (F1@50) and 0.75 (F1@75)—to evaluate detection performance under varying levels of strictness. The mean F1-score (mF1) is then calculated as

(10) $$mF_{1}=\frac{\sum_{i=10}^{19}F_{1}@(i\times 5)}{10}$$

For the TuSimple dataset [34], we employ three standard evaluation metrics, namely accuracy (Acc), false positive rate (FPR), and false negative rate (FNR). Accuracy is defined as

(11) $$Accuracy=\frac{\sum_{clip}C_{clip}}{\sum_{clip}S_{clip}}$$

where $C_{clip}$ and $S_{clip}$ represent the number of correctly predicted and ground truth lane pixels, respectively. A predicted lane is considered correct if more than 85% of its predicted points lie within a 20-pixel distance from the corresponding ground truth points. Given that TuSimple primarily focuses on highway scenarios with clear lane markings and minimal occlusion, using accuracy as the principal metric is appropriate as it effectively reflects detection performance in such structured environments.

### 4.4. Quantitative Evalution

Experimental results are summarized in Table 3 and Table 4, the former of which reports the performance of our method on the CULane dataset in comparison with existing approaches. Under a unified backbone setting using ResNet-18 [29], our model achieves F1@50 and F1@75 values of 79.82 and 62.45, respectively, setting a new state-of-the-art benchmark and outperforming SGNet by nearly 10 percentage points, highlighting substantial improvements in lane localization precision and overall detection accuracy. Compared to previous methods, our approach consistently outperforms in most challenging scenarios, with particularly notable gains in shadow (+2.44) and curve (+1.18) scenes relative to CLRNet [9]. These improvements signify a meaningful improvement on the established CULane [11] benchmark, demonstrating the proposed method’s enhanced robustness to complex road conditions, especially under varying illumination and curvature.

### 4.5. Qualitative Evalution

**Figure 7:**
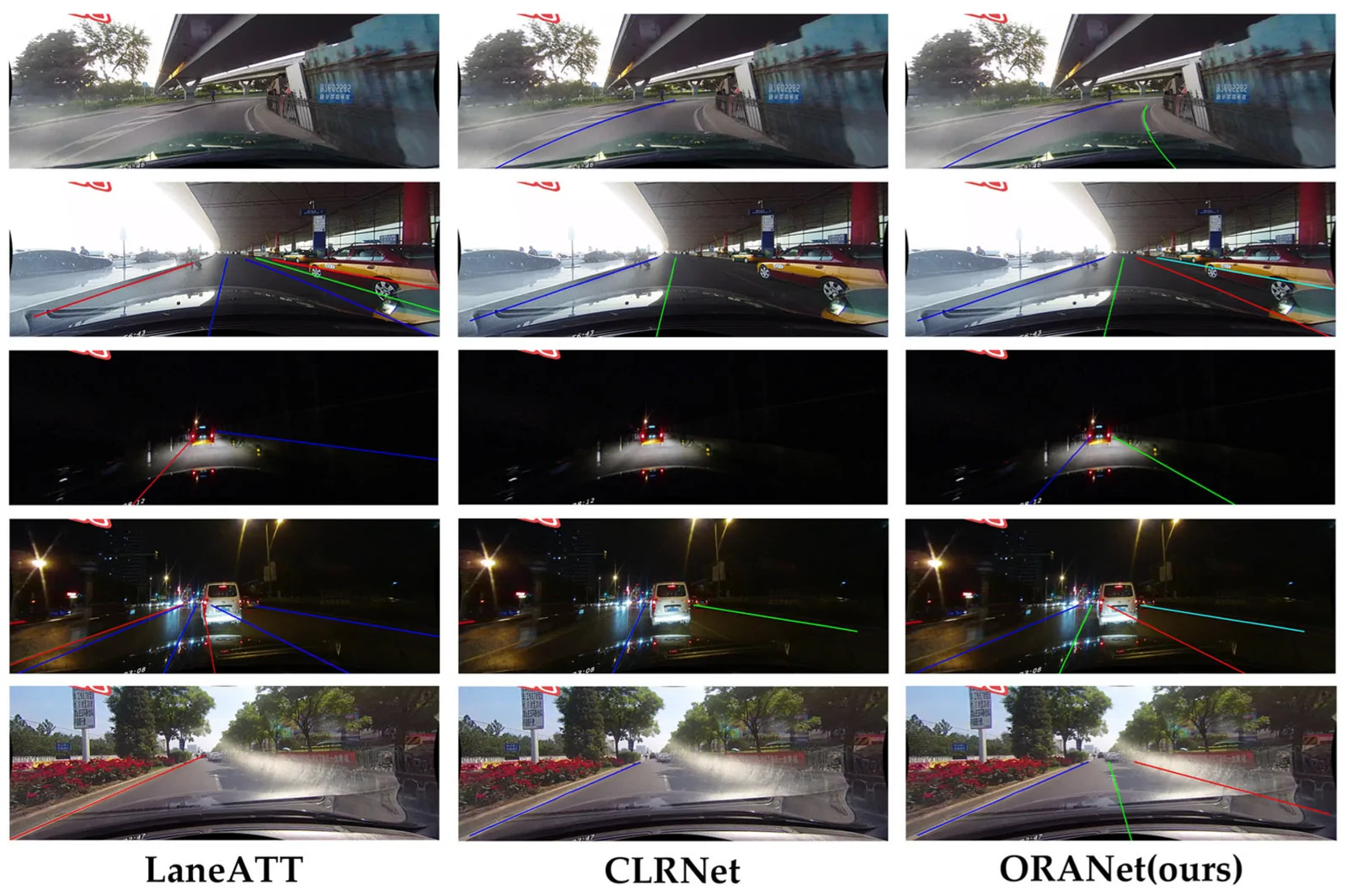
Qualitative evaluation results of our ORANet with LaneATT and CLRNet.

Qualitative comparisons among LaneATT [27], the baseline CLRNet [9], and our proposed ORANet are presented in Figure 7. Each row corresponds to a distinct driving scenario, such as curves, shadows, nighttime, glare, and compound conditions. From left to right, the columns show the results of LaneATT [27], CLRNet [9], and ORANet, respectively. These visualizations demonstrate that ORANet achieves more accurate and continuous lane localization, exhibiting greater stability and robustness under challenging conditions compared with the other methods.

**Figure 8:**
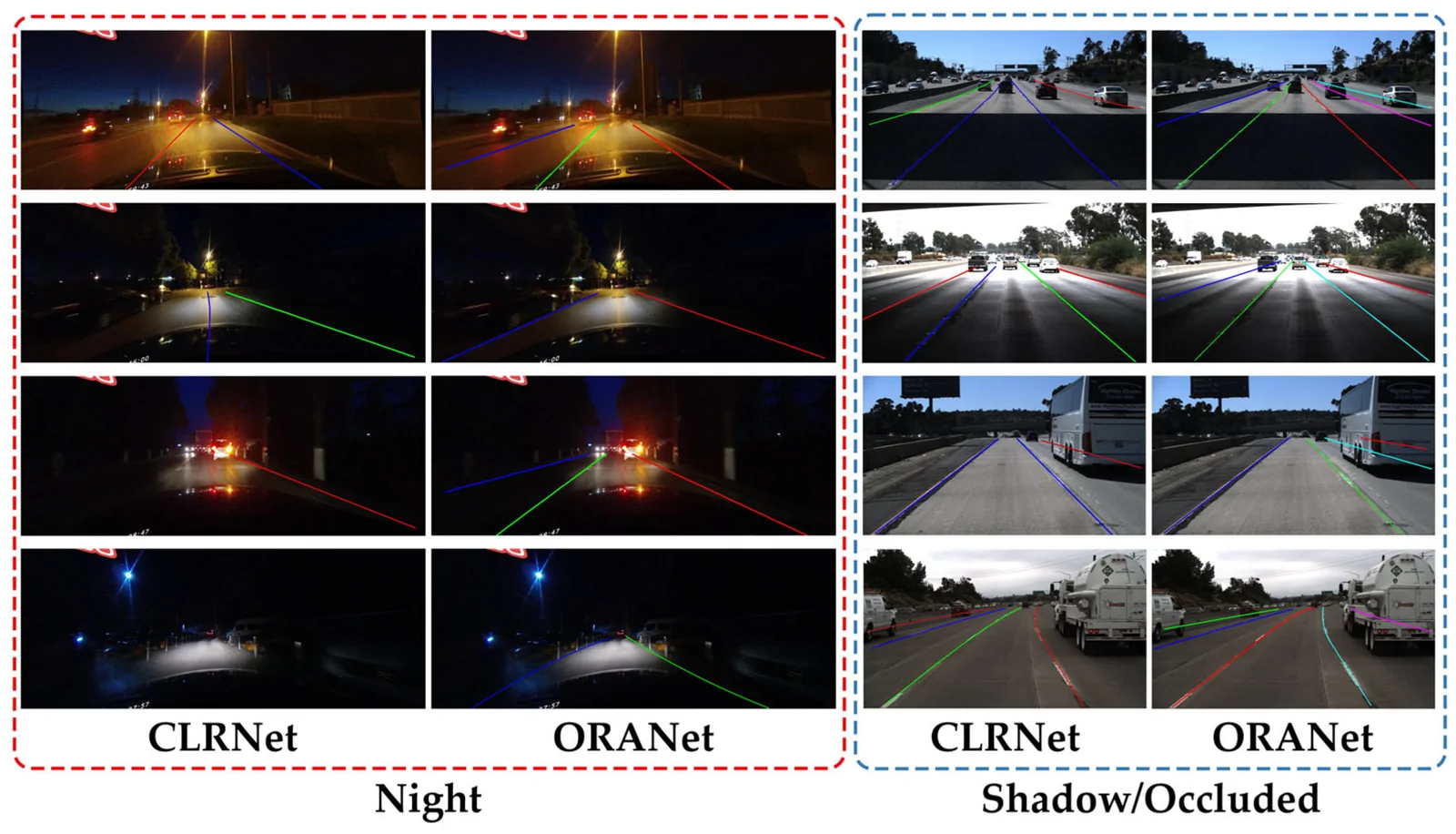
Qualitative results under night, shadow, and occlusion scenarios.

Furthermore, to further validate the advantages of our method under extreme conditions, additional qualitative results are illustrated in Figure 8. As shown, ORANet achieves superior performance in nighttime scenes, under-bridge shadow environments, and scenarios with severe occlusions caused by surrounding vehicles. In such challenging cases, CLRNet [9] often suffers from missed or false detections due to its limited ability to capture discriminative features under low illumination and incomplete visual cues. By contrast, ORANet effectively alleviates these issues through the joint contribution of CSSA and EnCA. Specifically, the channel shuffling operation in CSSA promotes inter-channel information exchange, enabling the network to suppress redundant activations and emphasize subtle but critical lane features. Meanwhile, EnCA enriches the representation of long-range structural dependencies, allowing the model to maintain lane continuity even when markings are partially invisible. Together, these mechanisms empower ORANet to accurately recover lane lines that CLRNet [9] fails to detect or misclassifies, thereby achieving more robust and reliable lane localization across adverse conditions.

### 4.6. Ablation Study and Analysis

We conducted detailed ablation studies on the CULane [11] and TuSimple [34] datasets to comprehensively evaluate the effectiveness of each proposed component, as summarized in Table 5. The incorporation of the EnCA module significantly enhances the network’s ability to model directional and long-range spatial structures of lanes while effectively integrating global semantic information across different layers. As shown in Table 5, our EnCA module (Row 3) achieves a noticeable performance lift over both the baseline (Row 1) and the conventional coordinate attention (CA) mechanism (Row 2), thus demonstrating its superior capability in capturing lane-specific contextual features. Similarly, employing the CSSA module alone (Row 5) yields improved accuracy over the baseline and also outperforms the widely used CBAM module (Row 4), a result that underscores the effectiveness of its channel shuffle mechanism in promoting superior inter-channel communication and enhancing local feature discriminability.

Most notably, when both EnCA and CSSA are jointly deployed (Row 6), the model achieves the best performance across both datasets, with an F1-score and accuracy of 79.82 and 96.97 on CULane and TuSimple, respectively. This synergy establishes a hierarchical enhancement pathway whereby CSSA first refines local feature representation, providing semantically rich and noise-reduced features for EnCA, which in turn aggregates robust global contexts. The complete framework effectively bridges local detail extraction and global structure reasoning, leading to a more robust feature representation for lane detection in complex environments.

To justify our architectural design and explicitly analyze the computational complexity, we conducted an ablation study on the key hyperparameters of our CSSA and EnCA modules. As detailed in Table 6, we evaluated variants with different complexity settings on the CULane validation set, reporting the F1-score, number of parameters (Params), computational cost (GFLOPs), and inference speed (FPS).

For the CSSA module, we investigated the channel reduction ratio. Compared to our chosen setting (Ratio: C/4), a heavier variant (C/2) increased Params and GFLOPs by approximately 15% and 16%, respectively, for a negligible gain in F1-score (+0.03). Conversely, a lighter variant (C/8) reduced model size and computation by about 9% and 10%, but at the cost of a more significant performance drop (−0.41 F1-score). For the EnCA module, replacing the efficient Depth-Wise Convolution (DWConv) with standard convolutions increased computational cost without any accuracy benefit, actually slightly lowering the F1-score.

This comprehensive complexity analysis validates that our final design (CSSA with C/4 ratio and EnCA with DWConv) achieves an optimal trade-off, delivering top performance with efficient resource utilization, as evidenced by the high FPS and balanced Params/GFLOPs.

A primary limitation of the proposed method is the computational cost incurred by the introduced modules. Although our design achieves a higher F1-score, it comes with an increase in model parameters and GFLOPs compared to the original baseline, as detailed in Table 6. This trade-off between accuracy and efficiency may constrain its deployment in resource-sensitive scenarios. In future work, we will explore model compression and lightweight architecture search to reduce this overhead.

We also investigated the integration of additional modules on top of EnCA and CSSA; however, this led to a decline in performance, likely due to excessive module stacking, which may cause the loss of critical feature details during extraction and thus diminish overall model effectiveness. This observation further highlights the necessity of maintaining a balanced and efficient design rather than simply increasing architectural depth or complexity.

Additionally, despite the overall performance improvement, the model remains susceptible to highly complex scenarios, such as intersections with missing lane markings. We plan to enhance robustness in these challenging environments by incorporating temporal information and synthesizing more diverse training data.

## 5. Conclusions and Discussion

This work introduces ORANet, an innovative framework for lane detection that emphasizes enhanced performance under complex road conditions. ORANet effectively overcomes limitations in feature representation and global feature fusion under challenging scenarios by introducing the EnCA and CSSA modules to strengthen the integration of slender lane structures with global semantics and to comprehensively enhance and refine local feature extraction, respectively.

Extensive evaluations of the CULane [11] and TuSimple [34] benchmarks validate the superiority of our approach. Notably, ORANet demonstrates substantially improved robustness under adverse conditions such as curves and nighttime, achieving nearly a three-percentage-point F1-score improvement over CLRNet [9] under shadowed scenarios, demonstrating exceptional capability in handling complex lane structures. Beyond challenging conditions, ORANet also breaks through performance bottlenecks on standardized datasets like TuSimple, attaining 96.95% accuracy. These advances come as a result of the innovative combination of EnCA and CSSA modules, establishing ORANet as a more reliable and precise solution for autonomous driving and ADAS.

While ORANet demonstrates strong performance across most scenarios—including challenging curves and shadowed conditions—limitations remain in environments lacking clear road references, such as unmarked intersections or areas without lane markings. In these cases, false positives and negatives may arise, possibly due to interference from grid lines or crosswalk patterns that confuse the model. Future work should therefore prioritize enhancing contextual understanding to better handle such edge cases and further improve detection robustness.

## Figures and Tables

**Table 1:** Summary of representative lane detection methods across four categories.

Category	Method	Dataset(s)	Backbone/Model	Feature Extraction Strategy	Evaluation Metrics	Validation/Findings
Segmentation-based	SCNN	CULane	VGG16	Spatial CNN for shape priors	F1, IoU	Improves structural continuity but computationally heavy
	ENet-SAD	CULane	ENet	Self-Attention Distillation	F1	Lightweight, enhances shallow features but less robust under occlusion
Row-wise-based	UFLD	TuSimple, CULane	ResNet	Row-wise classification, global features	Acc, FN, FP	Real-time speed, but limited local detail and sensitivity to tilt
	CondLaneNet	CULane	ResNet	Dynamic convolution, conditional anchors	F1, Acc	Handles occlusion better but high complexity
Keypoint-based	PINet	TuSimple	Hourglass	Keypoint estimation + clustering	Acc, FN, FP	Provides fine-grained lanes but requires costly post-processing
	FOLOLane	TuSimple, CULane	ResNet	Keypoint heatmaps + geometric constraints	Acc, FN, FP	Strong localization, but computationally expensive
Anchor-based	LaneATT	TuSimple, CULane	ResNet	Anchor-based with attention	Acc, FN, FP	Improves anchor-based detection but struggles with occlusion and curvature
	CLRNet	TuSimple, CULane	ResNet	Cross-layer refinement (FPN)	F1, Acc	Strong baseline, but lacks refined local feature extraction

**Table 2:** Effect of Learning Rate on CULane Validation Performance.

Learning Rate	F1@50 Score	Convergence Behavior
0.001	79.15	Unstable
0.0006	79.54	Stable, but suboptima
0.0003 (ours)	79.58	Optimal stability and performance
0.0001	79.31	Slow convergence

**Table 3:** Comparative results of various methods on the CULane dataset. Results are color-coded with red indicating optimal outcomes, green intermediate, and blue suboptimal.

Method	Backbone	mF1	F1@50	F1@75	Normal	Crowded	Shadow	No Line	Arrow	Curve	Cross	Night
SCNN [11]	VGG16	38.84	71.60	39.84	90.60	69.70	66.90	43.40	84.10	64.40	1990	66.10
RESA [31]	ResNet34	-	74.50	-	91.90	72.40	72.00	46.30	88.10	68.60	1896	69.80
FastDraw [37]	ResNet50	47.86	75.30	53.39	92.10	73.10	72.80	47.70	88.30	70.30	1503	69.90
E2E [34]	ERFNet	-	74.00	-	91.00	73.10	74.10	46.60	85.80	71.90	2022	67.90
UFLD [8]	ResNet18	38.94	68.40	40.01	87.70	66.00	62.80	40.20	81.00	57.90	1743	62.10
PINet [19]	Hourglass	46.81	74.40	51.33	90.30	72.30	68.40	49.80	83.70	65.20	1427	67.70
LaneATT [27]	ResNet18	47.35	75.13	51.29	91.17	72.71	68.03	49.13	87.82	63.75	1020	68.58
LaneAF [38]	DLA34	50.42	77.41	56.79	91.80	75.61	79.12	51.38	86.88	72.70	1360	73.03
SGNet [39]	ResNet18	-	76.12	-	91.42	74.05	72.17	50.16	87.13	67.02	1164	70.67
CLRNet [9]	ResNet18	55.23	79.58	62.21	93.30	78.33	79.66	53.14	90.25	71.56	1321	75.11
Ours	ResNet18	55.33	79.82	62.45	93.54	77.97	82.10	52.89	90.61	72.74	1333	75.59

**Table 4:** Comparative results of various methods on the TuSimple dataset. Results are color-coded with red indicating optimal outcomes, green intermediate, and blue suboptimal.

Method	Backbone	Acc (%)	FP (%)	FN (%)
SCNN [11]	VGG16	96.53	6.17	1.80
RESA [31]	ResNet34	96.82	3.63	2.48
PolyLaneNet [40]	EfficientNetB0	93.36	9.42	9.33
E2E [34]	ERFNet	96.02	3.21	4.28
UFLD [8]	ResNet18	95.82	19.05	3.92
UFLD [8]	ResNet34	95.86	18.91	3.75
LaneATT [27]	ResNet18	95.57	3.56	3.01
LaneATT [27]	ResNet34	95.63	3.53	2.92
LaneATT [27]	ResNet122	96.10	5.64	2.17
CLRNet [9]	ResNet18	96.84	2.28	1.92
Ours	ResNet18	96.97	3.14	1.42

**Table 5:** Results of Ablation experiment. Red numbers are the best.

Configuration	F1-CULane	Acc(%)-Tusimple
Baseline(CLRNet)	79.58	96.84
Baseline + CA	79.54	96.86
Baseline + EnCA	79.63	96.92
Baseline + CBAM	79.60	96.87
Baseline + CSSA	79.71	96.89
Baseline + EnCA + CSSA(Ours)	79.82	96.97

**Table 6:** Analysis of Module Complexity on CULane Validation Performance.

Module	Complexity Setting	F1-Score	Params(M)	GFLOPs	FPS
CSSA	High(Ratio:C/2)	79.79	23.5	21.5	105
CSSA	Low(Ratio:C/8)	79.41	18.5	16.7	137
EnCA	High(Standard Conv)	79.75	24.1	22.5	100
None	Original	79.58	19.8	17.4	129
Both	Ours(C/4; DWConv)	79.82	20.4	18.5	123

## Data Availability

The original contributions presented in this study are included in the article. Further inquiries can be directed to the corresponding author.
